# Reporting and evaluation of assumptions and certainty of evidence in network meta-analyses

**DOI:** 10.1017/rsm.2025.10045

**Published:** 2025-11-20

**Authors:** Kansak Boonpattharatthiti, Kanyaphak Chueadi, Phiyanuch Thimkorn, Deborah M. Caldwell, Nathorn Chaiyakunapruk, Teerapon Dhippayom

**Affiliations:** 1 The Research Unit of Evidence Synthesis (TRUES), Faculty of Pharmaceutical Sciences, Naresuan University, Thailand; 2 Faculty of Pharmaceutical Sciences, Burapha University, Thailand; 3 Abhaibhubejhr College of Thai Traditional Medicine, Prachinburi, Faculty of Public Health and Allied Health Sciences, Praboromarajchanok Institute, Ministry of Public Health, Thailand; 4 Population Health Sciences, Bristol Medical School, University of Bristol, UK; 5 Department of Pharmacotherapy, The University of Utah College of Pharmacy, USA; 6 IDEAS Center, Veterans Affairs Salt Lake City Healthcare System, USA

**Keywords:** assumption, certainty of evidence, cross-sectional, network meta-analysis

## Abstract

Network meta-analysis (NMA) facilitates the comparison of multiple treatments by integrating both direct and indirect evidence. Applications of NMA in medical decision making have grown exponentially. However, the validity of NMA findings depends on key assumptions: homogeneity, transitivity, and consistency. A lack of consistent assessment of these assumptions potentially compromises the reliability of NMA outcomes. The objective of this study is to evaluate the extent to which researchers address NMA assumptions and report the assessment of evidence certainty in NMA publications. A total of 22,079 studies were identified from PubMed, Embase, and Cochrane CENTRAL (January 2010–August 2024). A sample of 393 NMAs was calculated to represent this population and randomly selected. Data on study characteristics, NMA assumptions, and the certainty of evidence were extracted and analyzed descriptively. Of the 393 NMAs, 71.8% were published between 2020 and 2024. Homogeneity was assessed in 300 (76.3%) NMAs, transitivity in 45 (11.5%) NMAs, and consistency in 265 (67.4%) NMAs. The certainty of evidence was assessed in 110 (28.0%) studies, predominantly using GRADE (71 NMAs; 18.1%) or CINeMA (29 NMAs; 7.4%). NMAs published in journals with high-impact factors more frequently evaluate these aspects than those published in low-impact journals. The assessment of NMA assumptions is inconsistently reported across studies, particularly for transitivity and consistency assumptions. Our findings highlight the need for standardized protocols or reporting guidelines to ensure these assessments are conducted and transparently reported.

## Highlights

### What is already known?

- Network meta-analysis (NMA) allows for the simultaneous comparison of multiple treatments by integrating both direct and indirect evidence.
- The validity of NMA depends on three key assumptions: homogeneity, transitivity, and consistency.
- Evaluating the certainty of evidence is also critical to support decision making and strengthen confidence in NMA findings.

### What is new?

- This study analyzed 393 NMAs published between 2010 and 2024 to examine how well these studies assessed the three key assumptions and the certainty of evidence.
- While homogeneity (76.3%) and consistency (67.4%) were frequently assessed, transitivity was rarely evaluated (11.5%).
- Less than one third of NMAs assessed the certainty of evidence.
- NMAs published in high-impact journals were more likely to assess all three assumptions and the certainty of evidence than those in low-impact journals.

### Potential impact for RSM readers

- The findings highlight critical gaps in the evaluation and reporting of key NMA assumptions and certainty of evidence.
- To improve the transparency and reliability of NMA findings, standardized protocols should be adopted and refined to ensure these assessments are routinely conducted and clearly reported.
- Evidence synthesis researchers are encouraged to assess the certainty of evidence when conducting NMAs to enhance the applicability and credibility of their results.

## 1 Introduction

Network meta-analysis (NMA) is a statistical technique that allows the simultaneous comparison of multiple treatments by combining both direct and indirect evidence in a single analysis.^1^ NMA can provide the relative treatment effects between any pair of interventions and allows for the estimation of the ranking and hierarchy of interventions.^2^ Over the past decade, NMA has gained popularity in various medical fields and is estimated to grow exponentially.^3^ It has been used to inform medical decisions and derive treatment recommendations.^4^^,^ ^5^

The validity of NMA findings relies on several key assumptions, that is, homogeneity, transitivity, and consistency.^6^ The homogeneity assumption assumes that trials are sufficiently similar. In a fixed effect model, all trials are assumed to estimate the same treatment effect, while in a random effects model, treatment effects vary but follow a typical distribution.^6^ The transitivity assumption refers to the trials from the different comparisons are sufficiently similar for the moderator of relative treatment effect.^6^^,^ ^7^ The consistency assumption refers to the agreement between direct and indirect evidence regarding treatment effects.^2^ Failure to properly assess NMA assumptions can compromise the validity of NMA findings.^8^^,^ ^9^ A scoping review of methodologies used in NMAs of psychological interventions found that 90.4% of the included studies assessed the homogeneity assumption, while 89.8% assessed the consistency assumption.^10^

It remains unclear to what extent researchers assess the assumptions that underpin the validity of NMA. To address this gap, we examined how these assumptions were reported and performed in published NMAs. Additionally, we explored how the certainty of evidence derived from NMA findings is evaluated.

## 2 Methods

A cross-sectional study was conducted to examine the assessment of NMA assumptions in published NMA studies from the past decades. We searched three databases—PubMed, Embase, and Cochrane CENTRAL—covering the period from January 2010 to August 2024, using search terms such as “network meta-analysis,” “mixed-treatment comparisons,” “indirect comparison,” and “multiple treatment comparisons.” The full search strategy can be found in Appendix 1 (see Supplementary Material). A total of 22,079 records were identified after removing duplicate.

### 2.1 Sample

Our study focuses on nonhuman subjects, with no prior data available regarding the population variance or proportion. Consequently, we calculate the sample size needed for the known population, utilizing the following formula^11^: 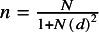 , where *N* is the total population (22,079) and *d* is the margin of error or precision (5%).

After applying the formula mentioned earlier, the required sample size for this study was determined to be 393 NMA studies. We randomly selected records from a total of 22,079 using a random number table generated in Microsoft Excel (Microsoft 365).

### 2.2 Study selection

K.C. screened the selected records based on the following criteria: studies must be NMAs related to humans in the clinical field, encompassing all published studies regardless of disease group. If a sampled NMA did not meet the eligibility criteria, K.C. continued the sampling process until a total of 393 eligible NMAs were obtained.

### 2.3 Data collection

Four experienced research assistants independently extracted data from all included studies, dividing the work into four equal parts, while K.B. and P.T. cross-checked the results. The extracted data included general characteristics such as the publication year, number of included studies in the NMA, number of participants included in the NMA, disease condition, reporting guideline, analytical framework, treatment arms in the NMA, and journal impact factors (JIF). Additionally, methodological characteristics of the included studies were extracted, including the approach used to assess NMA assumptions, such as homogeneity, transitivity, and consistency. Furthermore, we also extracted the certainty of evidence assessment. Disagreements between researchers were resolved through discussion, reaching a consensus with T.D.

### 2.4 Data analysis

We used descriptive statistics to analyze the extracted data. We charted the assessment of NMA assumptions and the certainty of evidence across the median journal impact factors, as identified by Journal Citation Reports (JCR) index for 2024,^12^ the year of publication, the reporting guideline, and other study characteristics. All analyses were conducted using Microsoft Excel (Microsoft 365).

## 3 Results

### 3.1 Characteristics of included NMA

A total of 393 NMAs were included in the study. Of these, 282 (71.8%) NMAs were published between 2020 and 2024, 97 (24.7%) NMAs between 2015 and 2019, and 14 (3.6%) NMAs between 2009 and 2014. The median number of trials included in the NMAs was 22 (IQR: 14–38), while the median number of participants was 4582 (IQR: 1891–10,208). The median JIF of the included NMAs was 3.3 (IQR: 2.40–5.05) (Table 1).

**Table 1:** General characteristic of included studies

Characteristics	Total (*n* = 393)	
	*N*	%
1. Year published		
2009–2014	14	3.6
2015–2019	97	24.7
2020–2024	282	71.8
2. Number of included study and participants		
Number of included trials in NMA, median (IQR)	22 (14–38)	
Number of included participants, median (IQR)	4582 (1891–10,208)	
Journal impact factors (JIF), median (IQR)	3.30 (2.40–5.05)	
3. Journal publications		
Journals with relatively higher JIFs	194	49.3
Journals with relatively lower JIFs	199	50.6
4. Disease condition		
Oncological disorders	69	17.6
Cardiovascular disorders	53	13.5
Neurological disorders	43	10.9
Gastrointestinal disorders	37	9.4
Rheumatological disorders	36	9.2
Others	155	39.4
5. Type of included studies		
Experimental study	362	92.1
Observational study	21	5.3
Observational study and experimental study	8	2.0
Not reported	2	0.51
6. Treatment arms in a network meta-analysis		
The number of treatment arms contributing to direct comparisons in a network meta-analysis, median (IQR)	11 (6–19)	
The number of treatment arms have more than two studies in the NMA, median (IQR)	5 (3–10)	
7. Reporting guideline used		
PRISMA NMA	148	37.7
PRISMA 2009	113	28.7
PRISMA 2020	42	10.7
PRISMA NMA and PRISMA 2020	2	0.5
PRISMA IPD	1	0.3
Not reported	87	22.1
8. Analytical framework		
Frequentist framework	196	49.9
Bayesian framework	183	46.6
Both Bayesian and frequentist framework	3	0.7
Not reported	8	2.1
Others	3	0.7

We used the median JIF of 3.3 as an operational cutoff point to classify the published NMAs into journals with relatively higher or lower JIFs. The resulting median JIFs for the higher or lower JIFs subgroups were 4.6 and 0.5, respectively. Among the 194 NMAs published in journals with relatively higher JIFs, 29 (7.4%) NMAs were published in journals with an impact factor greater than 10. The top five disease conditions studied in the included NMAs were oncological disorders (69 NMAs; 17.6%), cardiovascular disorders (53 NMAs; 13.5%), neurological disorders (43 NMAs; 10.9%), gastrointestinal disorders (37 NMAs; 9.4%), and rheumatological disorders (36 NMAs; 9.2%) (Table 1).

The most frequently included primary study design was experimental, either randomized controlled trials or quasi-experimentals (362 NMAs; 92.1%), followed by observational (21 NMAs; 5.3%). Only 8 (2.0%) NMAs included both observational and experimental study designs. The median number of treatment arms contributing to direct comparisons in the NMAs was 11 (IQR: 6–19), and the median number of treatment arms with more than two studies in the arm was 5 (IQR: 3–10) (Table 1).

Among the included NMAs, 148 (37.7%) reported adherence to the Preferred Reporting Items for Systematic Reviews and Meta-Analyses (PRISMA) extension for Network Meta-Analysis (PRISMA NMA). Additionally, 113 (28.7%) NMAs reported adherence to PRISMA 2009, while 42 (10.7%) NMAs reported adherence to PRISMA 2020. The frequentist framework was applied in 196 (49.9%) NMAs, whereas the Bayesian framework was utilized in 183 (46.6%) NMAs (Table 1).

### 3.2 Methodological assessment of NMA assumptions

A total of 300 (76.3%) NMAs of 393 assessed the homogeneity assumption using a statistical test for heterogeneity, while only 32 (8.1%) NMAs qualitatively assessed the clinical heterogeneity of the included studies. Transitivity was assessed by comparing the distribution of potential effect modifiers in 34 (8.7%) NMAs and by evaluating the similarity of the patient, intervention, comparator, outcome, and study design (PICOS) of the included studies in 11 (2.8%) NMAs (Table 2).

**Table 2:** Methodological assessment of NMA assumptions and certainty of evidence

Methodological assessment	Total (*n* = 393)	%
1.1 Homogeneity assumption assessment		
Using statistical test for heterogeneity	300	76.3
Assessing clinical heterogeneity of included studies	32	8.1
1.2 Transitivity assumption assessment		
Comparing the distribution of potential effect modifiers	34	8.7
Assessing the similarity of the PICOS of included studies	11	2.8
1.3 Consistency assumption assessment		
Node splitting	159	40.5
Global inconsistency model	44	11.2
Design by treatment interaction model	43	10.9
Single loop inconsistencies	43	10.9
Assessing model fit and deviance	14	3.6
Compare direct and indirect effect estimates	14	3.6
Q-statistics	12	3.1
Net heat plots	8	2.0
H statistics	4	1.0
*I*^2^ statistic	4	1.0
Assessing consistency using back calculation	3	0.8
Bayesian model	3	0.8
Others	10	2.5
2. Certainty of the evidence assessment		
GRADE approach	71	18.1
CINeMA (Confidence in Network Meta-Analysis)	29	7.4
Authors used their own criteria	10	2.5

A total of 265 (67.4%) NMAs assessed the consistency assumption. The methodological approach used to assess the consistency assumption varied across NMA studies. Among these, 159 (40.5%) NMAs used node-splitting approaches, 44 (11.2%) NMAs applied global inconsistency models, and 43 (10.9%) NMAs each employed the design-by-treatment interaction model and single-loop inconsistency methods. Additionally, 14 (3.6%) NMAs each assessed model fitness and deviance and compared direct and indirect effect estimates (Table 2).

### 3.3 Assessment of the certainty of evidence

A total of 110 (27.9%) NMAs assessed the certainty of evidence. Among these, 71 (18.1%) NMAs used the GRADE approach, 29 (7.4%) NMAs used the Confidence in Network Meta-Analysis (CINeMA) online platform, and 10 (2.5%) NMAs applied their own criteria (Table 2).

### 3.4 Assessment of NMA assumptions and evidence certainty by journal impact factor

Among the 194 NMAs published in high-impact journals, the homogeneity, transitivity, and consistency assumptions were assessed in 159 (82.0%), 33 (17.0%), and 131 (67.5%) NMAs, respectively. These assumptions were assessed in 141 (70.9%), 12 (6.0%), and 134 (67.3%) NMAs published in low-impact journals, respectively. Twenty-nine (14.9%) NMAs published in high-impact journals assessed all three NMA assumptions, while only 9 (4.5%) NMAs published in low-impact journals assessed these. In high-impact journals, 61 (15.5%) NMAs assessed the certainty of evidence using either the GRADE approach or CINeMA, compared to 40 (10.2%) in low-impact journals (Table 3).

**Table 3:** Comparison of methodological assessment and certainty of evidence assessment in network meta-analyses by study characteristics

					All three	GRADE	
	Total	Homogeneity	Transitivity	Consistency	assumptions	approach	CINeMA
Study characteristics	(*n* = 393)	*n* (%)^a^	*n* (%)^a^	*n* (%)^a^	*n* (%)^a^	*n* (%)^a^	*n* (%)^a^
Classified by the median of impact factor							
Journals with relatively higher JIFs	194	159 (82.0)	33 (17.0)	131 (67.5)	29 (14.9)	43 (22.2)	18 (9.3)
Journals with relatively lower JIFs	199	141 (70.9)	12 (6.0)	134 (67.3)	9 (4.5)	28 (14.1)	12 (6.0)
Classified by year of publication							
2009–2014	14	10 (71.4)	0 (0.0)	3 (21.4)	0 (0.0)	0 (0.0)	0 (0.0)
2015–2019	97	80 (82.5)	10 (10.3)	69 (71.1)	9 (9.3)	14 (14.4)	0 (0.0)
2020–2024	282	210 (74.5)	35 (12.4)	207 (73.4)	29 (10.3)	57 (20.2)	29 (10.3)
Classified by reporting guideline							
PRISMA NMA	148	113 (76.4)	24 (16.2)	115 (77.7)	18 (12.2)	32 (21.6)	16 (10.8)
PRISMA 2009	113	94 (83.2)	8 (7.1)	84 (74.3)	8 (7.1)	20 (17.7)	2 (1.8)
PRISMA 2020	42	33 (78.6)	5 (11.9)	25 (59.5)	3 (7.1)	7 (16.7)	4 (9.5)
PRISMA NMA and PRISMA 2020	2	2 (100.0)	0 (0.0)	1 (50.0)	0 (0.0)	0 (0.0)	1 (50.0)
PRISMA IPD	1	1 (100.0)	0 (0.0)	0 (0.0)	0 (0.0)	0 (0.0)	0 (0.0)
Classified by disease condition							
Oncological disorders	69	56 (81.2)	9 (13.0)	45 (65.2)	9 (13.0)	16 (23.2)	4 (5.8)
Cardiovascular disorders	53	41 (77.4)	4 (7.5)	29 (54.7)	1 (1.9)	6 (11.3)	1 (1.9)
Neurological disorders	43	34 (79.1)	4 (9.3)	33 (76.7)	4 (9.3)	7 (16.3)	5 (11.6)
Gastrointestinal disorders	37	29 (78.4)	2 (5.4)	21 (56.8)	2 (5.4)	4 (10.8)	2 (5.4)
Rheumatological disorders	36	23 (63.9)	3 (8.3)	26 (72.2)	2 (5.6)	3 (8.3)	1 (2.8)
Classified by type of included studies							
Experimental study	362	278 (76.8)	45 (12.4)	243 (67.1)	38 (10.5)	67 (18.5)	29 (8.0)
Observational study	21	16 (76.2)	0 (0.0)	14 (66.7)	0 (0.0)	2 (9.5)	1 (4.8)
Observational study and experimental study	8	4 (50.0)	0 (0.0)	6 (75.0)	0 (0.0)	1 (12.5)	0 (0.0)
Classified by analytical framework							
Frequentist framework	196	162 (82.7)	30 (15.3)	137 (69.9)	27 (13.8)	40 (20.4)	24 (12.2)
Bayesian framework	183	129 (70.5)	14 (7.7)	117 (63.9)	11 (6.0)	25 (13.7)	6 (3.3)
Both Bayesian and frequentist framework	3	3 (100.0)	0 (0.0)	3 (100.0)	0 (0.0)	1 (33.3)	0 (0.0)

a Percentages were calculated for each criterion.

### 3.5 Assessment of NMA assumptions and evidence certainty by year of publication

**Figure 1:**
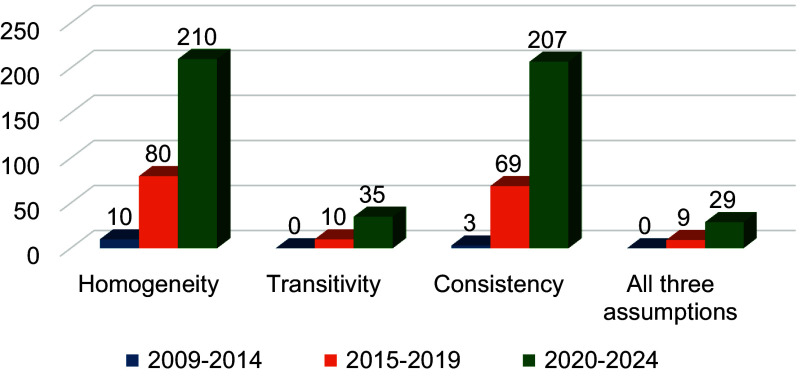
Comparison of methodological assessment by publication periods.

When categorizing the NMAs by year of publication, among 282 NMAs published between 2020 and 2024, 210 (74.5%) NMAs assessed the homogeneity assumption, 35 (12.4%) NMAs assessed the transitivity assumption, and 207 (73.4%) NMAs assessed the consistency assumption. Additionally, 57 (20.2%) NMAs assessed the certainty of evidence using the GRADE approach and 29 (10.3%) NMAs used CINeMA. Only 14 NMAs were published between 2009 and 2014. Of these, 10 (71.4%) NMAs assessed the homogeneity assumption and 3 (21.4%) NMAs assessed the consistency assumption. No NMAs assessed the transitivity assumption or the certainty of evidence during this period (Table 3). The assessment of all NMA assumptions increased over time, from zero in 2009–2014 to 29 studies in 2020–2024 (Figure 1).

### 3.6 Assessment of NMA assumptions and evidence certainty by reporting guideline

Among the 148 NMAs that reported adherence to PRISMA-NMA, the assumptions of homogeneity, transitivity, and consistency were assessed in 113 (76.4%), 24 (16.2%), and 115 (77.7%) NMAs, respectively. In contrast, among those that reported adherence to PRISMA 2020, these assumptions were assessed in 33 (78.6%), 5 (11.9%), and 25 (59.5%), respectively. The certainty of evidence was assessed using the GRADE approach in 32 (21.6%) NMAs, while 16 (10.8%) NMAs used CINeMA among those that reported adherence to PRISMA-NMA (Table 3).

### 3.7 Assessment of NMA assumptions and evidence certainty across other study characteristics

Among the included NMAs, those addressing oncological disorders were most likely to assess all three key assumptions (9 NMAs; 13.0%). Homogeneity and consistency were assessed in over half of the studies across all disease categories, whereas transitivity was rarely evaluated. The GRADE approach was used to assess certainty of evidence in 23.2% of oncological NMAs, followed by 16.3% in neurological, 11.3% in cardiovascular, 10.8% in gastrointestinal, and 8.3% in rheumatological disorders. CINeMA was used most frequently in neurological (11.6%) and oncological (5.8%) NMAs (Table 3).

In terms of study design, 10.5% of NMAs with experimental studies assessed all three assumptions, while none of the observational NMAs did so. Notably, transitivity was evaluated only in NMAs using experimental designs. GRADE and CINeMA were applied in 18.5% and 8.0% of experimental NMAs, respectively, compared to 9.5% and 4.8% of observational NMAs (Table 3).

Regarding analytical frameworks, all three assumptions were assessed more often in NMAs using the Frequentist framework (13.8%) than the Bayesian framework (6.0%). The GRADE approach was used in 20.4% of Frequentist NMAs and 13.7% of Bayesian NMAs, while CINeMA was applied in 12.2% and 3.3%, respectively (Table 3).

## 4 Discussion

The findings of this study highlight important patterns in how researchers assess key methodological assumptions in NMAs. While most NMAs used statistical methods to evaluate homogeneity, few considered clinical heterogeneity, highlighting the need for a more comprehensive approach. Without assessing clinical and methodological heterogeneity, the reasons for differences between studies remain unclear, limiting the interpretability of results. Additionally, the transitivity assumption was rarely assessed, raising concerns about validity, and consistency assessments varied widely in rigor. Most NMAs also failed to evaluate the certainty of evidence, potentially limiting the applicability of their findings.

The current assessments of NMA assumptions highlight inconsistencies in methodological approaches. This may be due to the lack of clarity and precise definitions of NMA-related terms and their interrelationships over the past 20 years.^4^ The homogeneity assumption is sometimes considered synonymous with similarity assumption.^13^ Moreover, the transitivity assumption, which assumes that all studies in the network have a similar distribution of effect modifiers, is closely related to both consistency and similarity assumptions.^4^ However, there remains no standard methods for evaluating these assumptions.

Our results suggest that studies reporting adherence only to PRISMA 2020 are less likely to assess transitivity and consistency compared to those following PRISMA-NMA. Even among studies claiming adherence to the PRISMA-NMA guidelines, only a small proportion—less than 15%—assessed all three assumptions. Although PRISMA-NMA is expected to encompass all three assumptions—homogeneity, transitivity, and consistency—these were often not adequately addressed, particularly transitivity. We would like to highlight the need for greater attention to this issue and recommend that newly submitted NMAs explicitly state their adherence to PRISMA-NMA and ensure that all key assumptions are appropriately evaluated. Furthermore, we suggest that journal editors and peer reviewers play a more proactive role by mandating the use of the PRISMA-NMA checklist during the submission and review process and by validating adherence as part of the manuscript evaluation criteria. These methodological variations further emphasize the lack of standardized practices across NMAs. A universally accepted framework with clear guidance on assessing homogeneity, transitivity, and consistency assumptions is needed to enhance the reliability of NMA findings. While PRISMA-NMA^14^ provide a reporting framework, it does not fully address these assumptions, leaving gaps in standardization. Refining reporting guidelines, such as PRISMA-NMA, to include explicit recommendations for evaluating these aspects would improve consistency.

This study has several limitations. First, although we systematically sampled NMAs from major databases, our selection was limited to published studies, potentially introducing publication bias, as unpublished or nonindexed NMAs may follow different methodological practices. Second, our sample size calculation did not reflect the entire population of published NMAs, but rather a broader set of studies—both eligible and ineligible—captured by our search strategy. As a result, the final sample of 393 included NMAs may exceed the actual number required to meet our intended precision, given that our sampling frame was larger than the true eligible population. Third, while we assessed the reporting of NMA assumptions, we did not evaluate their actual validity or the accuracy of the methods used, which may affect the interpretation of findings. For example, in cases where inconsistency was detected, we did not explore the process used to resolve it. Finally, our analysis relied on the information available in published articles, which may not fully capture the extent to which researchers internally assess these assumptions but choose not to report them. Further studies may be warranted to gain deeper insights into factors associated with the assessment of key assumptions.

## 5 Conclusion

While homogeneity is frequently evaluated, other assumptions remain underreported, potentially impacting the reliability of NMA findings. The limited assessment of evidence certainty further raises concerns about the transparency and applicability of NMA results. To ensure reliable NMA findings, it is crucial that all NMA assumptions are thoroughly evaluated and transparently reported. Our findings emphasize the need for standardized protocols or reporting guidelines to improve the consistency and quality of these assessments.

## Supporting information

Boonpattharatthiti et al. supplementary material Boonpattharatthiti et al. supplementary material

## Data Availability

Data sharing not applicable—no new data generated.

## Supplementary material

To view supplementary material for this article, please visit http://doi.org/10.1017/rsm.2025.10045.
