# Supplementary material for: Reporting and evaluation of assumptions and certainty of evidence in network meta-analyses
Source: Res Synth Methods. 2025 Nov 20;17(3):557–66. doi: 10.1017/rsm.2025.10045 (PMC13126206; doi:10.1017/rsm.2025.10045)
Supplement: Boonpattharatthiti et al. supplementary material [file S1759287925100458sup001.docx]

**APPENDICES**

**How Well Network Meta-Analyses Have Reported And Evaluated Assumptions And Certainty Of Evidence?**

**Table of contents**

[Appendix 1: Search strategies from January 2010 to August 2024 2](#_Toc204766542)

[1.1 PubMed 2](#_Toc204766543)

[1.2 Embase 2](#_Toc204766544)

[1.3 CENTRAL 3](#_Toc204766545)

# Appendix 1: Search strategies from January 2010 to August 2024

## 1.1 PubMed

| **Search number** | **Query** | **Results** |
| --- | --- | --- |
| 1 | "Network meta-analys*" | 11,628 |
| 2 | "Mixed-treatment comparisons" | 178 |
| 3 | "Indirect comparisons" | 1,440 |
| 4 | "Adjusted indirect comparisons" | 171 |
| 5 | "Multiple-treatments meta-analysis" | 62 |
| 6 | "Multiple treatment comparisons" | 83 |
| 7 | "Indirect treatment comparisons" | 186 |
| 8 | "Network Meta-Analysis"[Mesh] | 6,244 |
| 9 | OR/1-8 | 12,643 |

## 1.2 Embase

| **Search number** | **Query** | **Results** |
| --- | --- | --- |
| 1 | ‘network meta-analysis’/exp OR ‘network meta-analysis’ | 15,426 |
| 2 | ‘mixed-treatment comparisons’ | 308 |
| 3 | ‘indirect comparisons’ | 2,413 |
| 4 | ‘adjusted indirect comparisons’ | 373 |
| 5 | ‘multiple-treatment meta-analysis’ | 78 |
| 6 | ‘multiple treatment comparisons’ | 104 |
| 7 | ‘indirect treatment comparisons’ | 512 |
| 8 | ‘network meta-analys*’ | 15,706 |
| 9 | ‘network meta-analysis’/exp | 9,332 |
| 10 | OR/1-9 | 17,619 |

## 1.3 CENTRAL

| **Search number** | **Query** | **Results** |
| --- | --- | --- |
| 1 | Network meta-analys* | 3,606 |
| 2 | “Mixed-treatment comparisons” | 37 |
| 3 | “Indirect comparisons” | 689 |
| 4 | “Adjusted indirect comparisons” | 61 |
| 5 | “Multiple-treatment meta-analysis” | 48 |
| 6 | “Multiple treatment comparisons” | 20 |
| 7 | “Indirect treatment comparisons” | 70 |
| 8 | MeSH descriptor: [Network Meta-Analysis] explode all trees | 152 |
| 9 | OR/1-8 | 4,062 |
|  |  |  |
